# Effects of proton therapy on the tumor immune system and clinical implications: A review

**DOI:** 10.1002/pro6.70089

**Published:** 2026-08-21

**Authors:** Rundong Liu, Mei Tao, Min Fu, Yingjia Hu, Zhen Tao, Guangyuan Hu

**Affiliations:** ^1^ Department of Oncology Tongji Hospital, Tongji Medical College, Huazhong University of Science and Technology Wuhan Hubei China; ^2^ Department of Biostatistics University of Pittsburgh Graduate School of Public Health, Pittsburgh Pennsylvania USA

**Keywords:** bulk RNA‐sequencing, chimeric antigen receptor T cell, immunotherapy, peripheral blood immune cells, proton therapy, single cell RNA‐sequencing, tumor immune microenvironment

## Abstract

Proton therapy, an emerging radiation therapy technique, has gained significant attention owing to its unique physical properties and biological effects. This review aims to explore the impact of proton therapy on the tumor immune system and of its combination with other therapies. This review outlines the fundamental principles and oncological applications of proton therapy, followed by an analysis of its impact on the immune system—encompassing draining lymph nodes, peripheral blood immune cells, tumor cell immune responses, and the tumor microenvironment. We further discuss current combinatorial strategies with immunotherapy and/or chemotherapy, which point toward promising clinical directions.

## Graphical abstract

Proton therapy is a novel radiotherapy technique. We review four aspects of proton therapy: application in various tumors, effects on the tumor immune system, combination with other therapies, and future challenges. By presenting these aspects in this review, we aim to inspire clinical interventions.

**Abbreviations**
ALC: absolute lymphocyte count
APA: anaplastic pituitary adenoma
BC: breast cancer
CAR‐T: chimeric antigen receptor T cell
CPT: conventional proton therapy
CTLA4: cytotoxic T‐lymphocyte associated protein 4
DFS: disease‐free survival
DSBs: DNA double‐strand breaks
ECs: endothelial cells
EDIC: effective radiation dose to immune cells
EndMT: endothelial‐to‐mesenchymal transition
ENI: elective nodal irradiation
GBM: glioblastoma
HCC: hepatocarcinoma
HIF1α: hypoxia‐inducible factor 1α
HNSCC: head and neck squamous cell carcinoma
HPBLs: human peripheral blood lymphocytes
ICBs: immune checkpoint blockades
IFN‐γ: interferon‐γ
IL1β: interleukin‐1β
TNFα: tumor necrosis factor‐α
IL‐6: interleukin‐6
IMPT: intensity‐modulated proton therapy
ITD: integral total dose volume
LAG3: lymphocyte activation gene‐3
LET: linear energy transfer
MBD: mean body dose
MDSCs: myeloid‐derived suppressor cells
MHD: mean heart dose
MID: mean liver dose
MLD: mean lung dose
MSLN: mesothelin
NF‐κB: nuclear factor‐κB
NPC: nasopharyngeal carcinoma
NSCLC: non‐small cell lung cancer
ORR: overall response rate
OS: overall survival
PDAC: pancreatic ductal adenocarcinoma
PD1: programmed cell death 1
PD‐L1: programmed cell death‐ligand 1
pFLASH: proton FLASH‐radiation therapy
pMBRT: proton minibeam radiotherapy
RBE: relative biological effectiveness
RIL: radiation‐induced lymphopenia
Tregs: regulatory T‐cells
STAT3: signal transducer and activator of transcription 3
TILs: tumor‐infiltrating lymphocytes
TIME: tumor immune microenvironment
TME: tumor microenvironment
CD8^+^ TRM: tissue resident memory cytotoxic
cDC1: conventional dendritic cells type 1
CD8+ TAMs: CD8+ tumor associated macrophages

## 1 INTRODUCTION

Proton therapy is an advanced form of radiation therapy that precisely targets tumors while minimizing damage to surrounding healthy tissues. Its main advantages are derived from its unique physical properties, with the Bragg peak being the most notable.^1^ Over the past few decades, the clinical applications of proton therapy have significantly progressed. In 2019, Yuan et al. summarized the application of proton therapy in treating head and neck squamous cell carcinoma (HNSCC), breast cancer (BC), non–small cell lung cancer (NSCLC), hepatocarcinoma (HCC), and prostate cancer.^2^ Furthermore, the pediatric,^2^ old,^3^ and pregnant^4^ patients may benefit the most from proton therapy for certain types of cancers.

The tumor immune system plays a crucial role in tumor progression and response to therapy.^5^ In this review, we describe the draining lymph nodes, peripheral blood immune cells, immune response of tumor cells, and the tumor immune microenvironment (TIME). (i) Proton therapy reduces radiation exposure to the draining lymph nodes, which are the primary sites for antigen presentation and T cell priming, which in turn better preserves the anti‐tumor immunity of the body. (ii) In peripheral blood immune cells, proton therapy exerts low irradiation toxicity, leading to a more pronounced active immunotherapy response. (iii) Stronger immunogenicity is triggered by proton therapy, which enhances the immune response of tumor cells. (iv) In the TIME, proton therapy builds a complicated immune landscape comprising various immune cells, cytokines, chemokines, and immune checkpoints that interact dynamically with tumor cells. The interaction among these elements can either promote tumor growth or enhance anti‐tumor immunity, highlighting the requirement for further research on the TIME.^6^, ^7^, ^8^, ^9^, ^10^ Therefore, understanding the evolution of the tumor immune system in response to proton therapy is essential for developing effective treatment strategies and improving patient outcomes.

The combination of proton therapy with immunotherapy and/or chemotherapy has gradually been utilized for the treatment of HNSCC, NSCLC, esophageal carcinoma, lymphoma, and pancreatic cancer. This review comprehensively describes the advantages and disadvantages of combination therapies for various tumor types. However, owing to the lack of sufficient clinical evidence, conducting large‐scale multi‐center prospective clinical trials is a direction for further study.

This review aims to provide an overview of the current understanding of proton therapy, particularly its effects on the tumor immune system, and related therapeutic strategies. In addition, we summarize bulk RNA sequencing for proton therapy, emphasizing the necessity for single‐cell RNA sequencing in future research.

## 2 BASIC PRINCIPLES OF PROTON THERAPY

### 2.1 Physical characteristics of proton therapy

Radiation therapy, an important cancer treatment strategy, is primarily classified into photon and particle therapies based on the type of radiation used. Proton therapy is a form of particle therapy that uses protons instead of conventional photons.^11^ The fundamental principle underlying proton therapy involves the unique physical properties of protons, particularly their ability to deliver energy to a specific depth within the tissue, known as the Bragg peak.^1^ The energy deposition pattern of proton is unique, characterized by a sharp increase in the dose at the Bragg peak, followed by a rapid fall‐off. This sharp dose gradient allows for precise dose distribution, helping eliminate tumors while sparing adjacent healthy tissues. Therefore, proton therapy is particularly suitable for tumors with complex anatomical relationships such as nasopharyngeal carcinoma (NPC).^12^ Furthermore, the linear energy transfer (LET) of protons is higher than that of photons, resulting in more complicated DNA double‐strand breaks (DSBs), which are difficult for tumor cells to repair.^13^ In addition, the relative biological effectiveness (RBE) of proton therapy is typically high,^14^ indicating that proton therapy requires a lower dose than photons to kill cancer cells. Recent studies have found that the relatively low risk of radiation‐induced lymphopenia (RIL) during proton therapy may be attributed to a smaller radiation field size and lower radiation dose.^15^

### 2.2 Comparison of proton therapy with conventional radiotherapy

Proton therapy has three major advantages over conventional radiotherapy. (i) Reduced radiation exposure: Proton therapy significantly decreases radiation exposure to healthy tissues, which is especially beneficial to pediatric populations, who are at a higher risk of long‐term side effects from radiation. Patients with medulloblastoma who received proton therapy reportedly exhibit improved neurocognitive outcomes and fewer secondary malignancies than those treated with traditional photon therapy.^16^, ^17^ (ii) Precise irradiation: Proton therapy delivers higher doses to tumors while minimizing exposure to the surrounding organs at risk, improving local control rates in various malignancies such as HNSCC, central nervous system cancer, and prostate cancer.^2^ (iii) Reduced radiation‐induced immunosuppression: Radiation‐induced immunosuppression significantly hinders the effectiveness of anti‐tumor treatments, and proton therapy alleviates this condition in NSCLC and HNSCC.^18^, ^19^ Notably, the mechanisms by which protons induce tumor cell death differ from those associated with photon therapy, leading to distinct phenotypes. This has been thoroughly reviewed by Nikitaki et al.^13^ Despite these advantages, the high cost of proton therapy facilities and limited availability of treatment centers remain the main barriers to its widespread adoption.^20^

### 2.3 Applications of proton therapy in different tumor types

The idea of utilizing protons for cancer treatment was first proposed by Robert R. Wilson^21^ in 1946, and the first patients received treatment in 1954.^22^ Subsequently, proton therapy was used to treat various tumor types because of its versatility and effectiveness.^2^ In recent years, proton therapy for HNSCC has shown promising results in reducing irradiation‐induced toxicities, thereby improving patients’ quality of life.^23^ In 2025, proton therapy was also used to treat head and neck mucosal melanoma, conjunctival malignant melanoma, and endometrial cancer.^24^, ^25^, ^26^, ^27^ Additionally, proton therapy has been investigated for its potential in treating primary hepatic and pancreatic cancers, in which its dosimetric advantages can lead to improved outcomes.^28^ Notably, in addition to eliminating the first malignancy, proton therapy also showed potential to prevent a second malignancy, which is one of the most serious late effects of radiation therapy.^29^ For example, a retrospective cohort study involving nine pediatric patients with low‐grade brain tumors indicated that proton therapy was associated with lower incidence of second malignancy and mortality, compared with photon therapy at the same dosage.^30^ Further research suggests that proton therapy could benefit patients with brain tumors because it can bypass traditional oxygen dependence.^31^ Several reviews and clinical studies have identified the role of proton therapy in the treatment of lung cancer,^32^, ^33^ triple‐negative breast cancer,^34^ and esophageal cancer.^35^ As clinical evidence continues to accumulate, the role of proton therapy in the treatment of various malignancies is expected to expand, with ongoing research targeting the optimization of treatment protocols and exploring combination therapies to enhance efficacy.^2^ Notably, proton irradiation induces distinct transcriptional profiles in various tumors, thereby shedding light on the underlying mechanisms (Table 1).

**TABLE 1:** Studies on Transcriptional Profiles* of Tumors after Proton Irradiation.

GEO ID	Tumor Types	Researcher	Publication Year
GSE149023	Breast Cancer	Cammarata et al.^36^	2020
GSE116325	Breast Cancer	Bravatà et al.^37^	2019
GSE103472	Breast Cancer	Bravatà et al.^38^	2018
GSE162986	Glioblastoma	Bravatà et al.^39^	2021
GSE127989	Glioblastoma	Cammarata et al.^40^	2019
GSE192817	Glioblastoma, Head and Neck Cancer, Prostate Cancer	Schniewind et al.^41^	2022
GSE90761	Head and Neck Cancer	Lupu‐plesu et al.^42^	2017
GSE45609	Lung Cancer	Beheshti et al.^3^	2014
GSE107444	Pancreatic Cancer	Fujinaga et al.^43^	2019

*Source: Gene Expression Omnibus (GEO) Database

## 3 IMPACT OF PROTON THERAPY ON DRAINING LYMPH NODES

Draining lymph nodes are important sites of immune response. They contain large numbers of T‐, B‐, and antigen‐presenting cells, which play crucial roles in tumor therapy.^44^, ^45^, ^46^, ^47^ During radiotherapy, immunogenic cell death and cytoplasmic DNA activate cGAS‐STING, which drives the recruitment and cross‐priming of dendritic cells in tumor‐draining lymph nodes. Subsequently, chemokines guide lymphocyte trafficking and expansion, ultimately leading to lymphocyte infiltration into tumors.^48^, ^49^ Irradiation of draining lymph nodes during radiotherapy may damage the immune system. For example, elective nodal irradiation (ENI) could reduce the expression of chemokines CXCR3 and CCR5, and inhibit the infiltration of IFNγ^+^/TNFα^+^ CD8^+^ T cells.^50^ In head and neck tumors, ENI reduces the number of CD69^+^CD8^+^ and CCR7^+^CD4^+^ T cells in the blood. It also decreases the number of LFA‐1^+^CD44^+^CD4^+^ T cells in the draining lymph nodes and tumor microenvironment (TME). These changes suppress antitumor immunity, thereby increasing the risks of both local tumor recurrence and distant metastasis.^51^ Notably, lymph node irradiation can also reduce the abscopal effect by inhibiting stem‐like CD8^+^ T‐cell proliferation within the tumor.^52^ Proton therapy, which is characterized by reduced irradiation exposure of lymph node, preserves anti‐tumor immunity. Using a mouse model of glioblastoma (GBM), Pham et al. found that proton therapy can reduce radiation exposure to lymph nodes, thereby preserving specific T‐cell subsets, such as naive CD8^+^ T cells.^53^ The effect of proton therapy on draining lymph nodes should be the focus of future studies, considering the limited number of studies.

## 4 IMPACT OF PROTON THERAPY ON PERIPHERAL BLOOD IMMUNE CELLS

### 4.1 Proton therapy relieves RIL and reduces the effective radiation dose to immune cells (EDIC)

RIL is an important biomarker for radiation‐induced immunotoxicity caused by exposure to radiation. RIL occurs when circulating lymphocytes pass through irradiated areas and is associated with poor survival outcomes in several solid tumors.^54^ Notably, unlike typical radiation‐induced cell death via apoptosis,^55^ human peripheral blood lymphocytes (HPBLs) exhibit a different mode of death during proton therapy.^56^ As observed by Miszczyk J et al., photons tended to induce necrosis rather than apoptosis in HPBL, especially at relatively high doses (≥ 1.5 Gy).^56^ Furthermore, photons could kill tumor cells through necrosis, which enhances immunogenicity and thereby improves abscopal effects.^56^, ^57^, ^58^ Proton therapy can reduce the occurrence of RIL via several mechanisms. These include reducing the radiation dose,^15^ minimizing the radiation field,^15^ and creating a volume effect by sparing larger volumes of normal tissue during Bragg peak therapy.^59^ For example, a cohort study of 143 patients with unresectable HCC showed that compared with those who did not receive proton therapy, those receiving proton therapy had a higher median absolute lymphocyte count (ALC) nadir (0.41 vs. 0.32 k/µL, *P* = 0.002). Furthermore, compared with patients treated with photon therapy, these patients had longer median overall survival (OS) (33.2 *vs*. 13.2 months, *P* = 0.002) and median disease‐free survival (DFS) (36.2 vs. 19.6 months, *P* = 0.018). These improvements may be attributed to the reduced radiation exposure at lymphocyte generation sites, such as the spleen.^60^ In addition, by reducing the radiation dose at non‐immune organs, proton therapy can alleviate lymphocytopenia and ultimately improve survival outcomes as well, which has been proved in patients with stage III NSCLC.^61^, ^62^

The EDIC model assesses the average radiation dose absorbed by circulating lymphocytes. It performs better in reflecting the immunotoxicity caused by irradiation. The EDIC model was proposed by Jin et al. as the Jin model^63^ and later modified into the Ladbury model by Ladbury et al.^64^ Recently, several studies have reported that proton therapy is efficient in reducing EDIC, which can ultimately improve tumor prognosis. For locally advanced NSCLC, proton therapy resulted in a significantly lower median EDIC than photon therapy (4.0 Gy vs. 5.3 Gy, *P*<0.001).^65^ Intensity‐modulated proton therapy (IMPT), an emerging proton therapy technique, exhibits excellent ability to minimize EDIC. For NSCLC, compared with intensity‐modulated radiotherapy, IMPT showed robustness in EDIC reduction (Jin model: 3.04 Gy vs. 4.99 Gy, *P* < 0.001; Ladbury model: 4.50 Gy *vs*. 7.60 Gy, *P* < 0.002), which led to an increase in the 2‐year OS rate (median 71% vs. 63%, *P* = 0.03).^66^ Considering the complicated calculation of EDIC, which includes the mean heart dose (MHD), lung dose (MLD), liver dose (MID), integral total dose volume (ITD), mean body dose (MBD), and so on,^63^, ^64^, ^67^ identifying the main driving factors is essential. For instance, a clinical study involving 10 patients with mediastinal Hodgkin lymphoma confirmed that the primary drivers of IMPT‐mediated EDIC reduction were integral dose reduction, accounting for 53.7%, and lung sparing, contributing 33.4%.^68^ These findings, along with those of Nakamura et al., highlight the significant role of the lungs in lymphocytopenia.^62^, ^68^ Additionally, tumor location is an important factor influencing IMPT‐mediated EDIC reduction. For locally advanced BC, integral dose reduction was the primary driver of IMPT‐mediated EDIC reduction and was associated with liver‐sparing and lung‐sparing in patients with right‐sided and left‐sided BC, respectively.^67^ In summary, personalizing the evaluation of the factors driving IMPT‐mediated EDIC reduction is indispensable.

### 4.2 Unique immune cell subpopulations and cell interactions

Cancer‐related inflammation plays a vital role in the initiation and progression of tumors. The key mechanisms include the intrinsic (oncogene activation) and extrinsic (inflammation or infection) pathways. Tumor‐related transcription factors such as nuclear factor‐κB (NF‐κB), signal transducer and activator of transcription 3 (STAT3), and hypoxia‐inducible factor 1α (HIF1α) play crucial roles. In addition, inflammatory mediators, such as cytokines, chemokines, and cyclooxygenase 2; myelomonocytic lineage cells (neutrophils and monocytes); and the cancer‐related inflammatory microenvironment are involved.^69^ Proton irradiation induces distinct alterations in immune cell subpopulations. The ratio of circulating neutrophils to lymphocytes, normally ranging from 0.78 to 3.53.^70^, is positively associated with poor progression‐free survival (PFS) and OS in HNSCC (PFS: 39.2% *vs*. 75.8%, *P*<0.001; OS: 50.9% *vs*. 83.8%, *P*<0.001), especially in nasopharyngeal, hypopharyngeal, and laryngeal cancers.^71^ Subsequently, Pham et al. conducted a detailed analysis of immune cell subpopulations. They classified leukocytes into T cells, B cells, NK cells, neutrophils, and monocytes and examined changes in each population separately. After 12 h of proton irradiation (4 fractions of 2.5 Gy), lymphocyte counts (CD4^+^ T cells, CD8^+^ T cells, B cells, and NK cells) did not decrease significantly, whereas neutrophil counts increased significantly (*P* = 0.03). In addition, they analyzed the effects of radiation parameters (irradiation type, irradiation volume, and dose rate) on six leukocyte subpopulations (including CD4^+^T cells, CD8^+^T cells, B cells, NK cells, neutrophils, and monocytes) using linear regression and tree‐based models, and observed that the impact of irradiation type on lymphocytes was stronger than that on myelocytes (CD8^+^ T cells were the most affected, followed by NK cells, B cells, and CD4^+^T cells).^72^ Similarly, a mouse‐based study showed that early proton irradiation increased myeloid cell subpopulations. Its effect on decreasing the number of lymphocytes was relatively delayed and minor.^73^ Furthermore, proton FLASH–radiation therapy (pFLASH) can up‐regulate the proportion of specific lymphocyte subsets at certain times. In a rat model of orthotopic glioma, Iturri et al. discovered that the proportion of B cells in the pFLASH group 24 hours after irradiation (257 ± 2 Gy/s) was significantly higher than that in the unirradiated group (*P* = 0.0054) and in the pFLASH group after 7 days of irradiation (*P*<0.0002).^74^ Further research has found that, compared with photon therapy, proton therapy can also avoid the loss of T cell receptor diversity.^75^ Proton irradiation protects lymphocyte subpopulations not only by preserving their numbers but also by enhancing interactions among specific subpopulations. For example, under the Bayesian network model, interactions between CD4^+^ T cells and CD8^+^ T cells, as well as between CD8^+^ T cells and B cells were strengthened after proton irradiation.^76^ Because these studies mainly focused on mice rather than humans, the applicability of these results in clinical practice must be carefully evaluated.

## 5 IMPACT OF PROTON THERAPY ON TUMOR CELL IMMUNE RESPONSE

The immune response of tumor cells plays a key role in tumor radiation therapy. Boopathi et al. concluded that cancer cells killed by radiation therapy could trigger both innate and adaptive immune responses to help eliminate tumors.^77^ Although proton therapy kills cells differently from photons and X‐rays,^55^, ^56^ the immune responses they induce may be similar at certain times.^78^ Du et al. exposed the esophageal cancer cell line KYSE450 to X‐rays, protons, and carbon ion beams at a dose of 15 GyE/fraction. After 3 days of irradiation, they observed shared interferon and innate immune responses, including those related to interferon signaling and cytokine production regulation, which were dependent on the STING–STAT1 axis.^78^ Furthermore, proton therapy failed to improve the clinical outcomes of NSCLC through radiation‐induced tumor immunity.^79^ Nevertheless, it is unwise to conclude that proton therapy offers no benefits for immune response and tumor control, as previous studies were limited by their in vitro settings and small sample sizes. Proton therapy has a greater immunogenic effect than conventional photon therapy in olfactory neuroblastoma and NPC, which may enhance antitumor immune responses and the effectiveness of immunotherapeutic drugs.^80^ In conclusion, rigorous in vivo and in vitro experiments should be conducted to clarify the effects of proton therapy on the immune responses of tumor cells.

## 6 IMPACT OF PROTON THERAPY ON THE TIME

**FIGURE 1:**
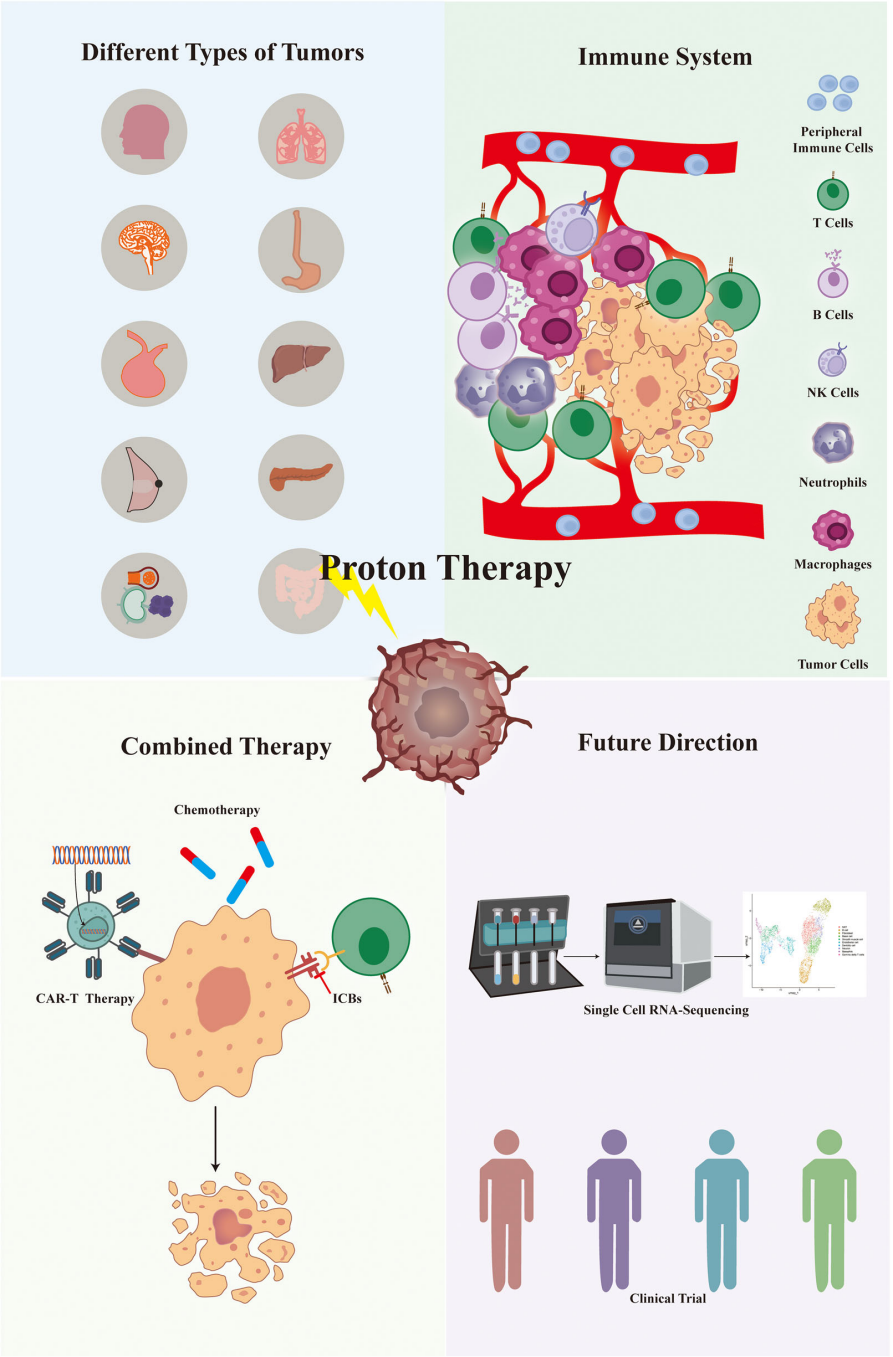
Landscape of the TIME.

In this section, we review the impact of proton therapy on the TIME from three perspectives: (i) infiltrating immune cells, (ii) cytokines and chemokines, and (iii) immune checkpoints. Figure 1 shows the TIME profile, and Table 2 presents the impact of proton therapy.

**TABLE 2:** Alteration of the TIME after proton therapy.

Tumor Types	Radiotherapy Strategy	Changes in the TIME	References
COAD	7 days after 16.4 Gy	CD8^+^ T cells, CD8^+^ T cells, TAM1s increased	^81^
		Tregs decreased	
COAD	7 days after 8 Gy	CD8^+^ T cells, CD8^+^PD1^+^T, NK cells decreased	^82^
		CD4^+^PD1^+^T, MDSCs increased	
NSCLC	8 days after 60 Gy/s	CD8^+^ T cells, TAM1s increased	^83^
		Tregs, TAM2s decreased	
HNSCC	12 hours after 5 Gy	IFN‐γ increased	^84^
		Exosomes decreased	
HCC	2 days after 3 Gy	CD8+ T cells increased	^85^
	2 days after 6 Gy	MDSCs, TAM2s increased	
	3 days after 12 Gy	IL‐6 increased	
Glioma	8 days after 257 ± 2 Gy/s	CD4^+^ T cells, CD8^+^ T cells, Tregs, CD8^+^ TRMs, NK cells, B cells increased	^74^
Medulloblastoma	5 days after 100 Gy/s	CD8^+^ T cells, TAM1s increased	^86^
		TAM2s decreased	
GBM	7 days after 30 Gy	CD8^+^ TRMs, B cells, CD8^+^ TAMs, cDC1 increased	^87^

Abbreviations: TIME, tumor immune microenvironment; COAD, colon adenocarcinoma; NSCLC, non‐small cell lung cancer; HNSCC, head and neck squamous cell carcinoma; HCC, hepatocellular carcinoma; GBM, glioblastoma; Tregs, regulatory T‐cells; CD8^+^ TRMs, CD8^+^ tissue resident memory cytotoxic T cells; NK cells, natural killer cells; TAMs, tumor associated macrophages; MDSCs, myeloid‐derived suppressor cells; cDC1, conventional dendritic cells type 1; PD‐1, programmed cell death 1; IFN‐γ, interferon gamma; IL‐6, interleukin‐6.

### 6.1 Impact on infiltrating immune cells

Photon therapy is a double‐edged sword for anti‐tumor immunity within the TME.^88^, ^89^ Recent studies have focused on the influence of proton therapy on immune cells within the TME. For instance, a study involving BALB/c mice with CT26 colon tumors demonstrated that proton irradiation led to increased immune cell infiltration, particularly at 7 and 14 days post‐treatment. RNA sequencing revealed the upregulation of genes associated with immune response and interferon signaling, suggesting that proton therapy not only induces direct tumor cell death, but also modulates the immune landscape, promoting a more favorable environment for immune activity.^81^ Similarly, Iturri et al. found that proton minibeam radiotherapy (pMBRT) enhanced antigen presentation and immune memory formation in a rat model of glioblastoma. Moreover, pMBRT significantly increased the density of tissue‐resident memory CD8^+^T cells (CD8^+^TRMs), B cells, conventional dendritic cells type 1 (cDC1), and CD8^+^ tumor‐associated macrophages (CD8^+^ TAMs).^87^ A subsequent study indicated that the preservation of specific immune cell subpopulations may result from proton therapy reducing the radiation exposure to the lymph nodes.^53^ However, in the HCC model, although proton therapy decreased the tumor size, it also recruited immune suppressive cells such as CD163^+^ M2 and myeloid‐derived suppressor cells (MDSCs) into the TME.^85^ Likewise, proton radiation triggered a stronger immunosuppressive response in colon tumors, manifested as an increase in MDSCs and CD4^+^PD1^+^T cells and a decrease in NK and CD8^+^T cells.^82^ FLASH therapy, which has an excellent capacity to improve radiotherapy efficiency and reduce toxicity to normal tissues,^90^, ^91^ has been applied to proton therapy in the laboratory.^92^ In a lung cancer model, pFLASH (60 Gy/s) enhanced the infiltration of cytotoxic CD8^+^T cells and M1‐type iNOS^+^ macrophages within the TME compared with conventional proton therapy (CPT, 1 Gy/s). Additionally, pFLASH significantly decreased the proportions of FOXP3^+^ regulatory T cells (FOXP3^+^ Tregs) and M2‐type CD163^+^ macrophages. Together, these changes led to a more effective reduction in the lung tumor burden and inhibited tumor cell proliferation.^83^ Furthermore, Ni et al. discovered that pFLASH had a significant impact on TAM. Specifically, pFLASH eliminated lipid oxidase expression and reduced low‐density lipid oxidation. This decreased PPARγ activity and arginase 1 expression, thereby inhibiting immunosuppressive macrophage polarization.^86^ In a rat model of orthotopic glioma, the advantages of pFLASH (257 Gy/s ± 2) over CPT (4 Gy/s ± 0.02) were not in tumor control but in alleviating neuroinflammation‐induced memory damage and increasing the infiltration of tumor‐infiltrating lymphocytes (TILs, including CD4^+^T cells, CD8^+^T cells, Tregs, CD8^+^TRMs, NK cells, and B cells) in the TME 8 days after radiation.^74^ The effects of pFLASH on specific immune cells were complex. For example, compared to CPT, pFLASH treatment reduced Tregs in the TME.^83^ However, this reduction was not statistically significant in the orthotopic glioma rat model (*P*>0.05).^74^ The varied effects of proton therapy on tumor‐infiltrating immune cells may be due to two factors: (i) immune cell heterogeneity within the TME across tumors^93^ and (ii) heterogeneity of the radiation dose.^94^ Moreover, overemphasizing the effects of proton therapy on single‐type cells may be inappropriate, because the interactions of immune cells in the TME are complex and critical.^95^ Hypoxia causes immune suppression in the TME.^96^ However, oxygen supplementation did not reverse the immune suppression caused by proton therapy in glioma‐bearing rats.^31^, ^97^

### 6.2 Changes in cytokines and chemokines

Proton therapy has been linked to significant alterations in cytokine and chemokine levels within the TME. Proton therapy increases pro‐inflammatory cytokines such as interleukin‐6 (IL‐6), interleukin‐1β (IL1β), and tumor necrosis factor‐α (TNFα), which regulate immune responses and tumor progression. For example, a study on HCC indicated that after 3 days of proton therapy, the tumor volume decreased, which was accompanied by an increase in IL‐6, suggesting that a pro‐inflammatory environment may contribute to tumor control.^85^, ^98^ A recent study using a rat GBM model also revealed an increase in pro‐inflammatory IL1β, chemokine ligand 1, and TNFα 7 days after proton radiation.^87^ Furthermore, in patients with HNSCC, proton therapy has been found to decrease the production of exosomes that suppress immune function, thereby potentially enhancing overall immune surveillance against tumors.^84^ These changes in cytokine profiles highlight the key role of proton therapy in TIME remodeling, shaping a complex inflammatory and immune response against tumors.

### 6.3 Regulation of immune checkpoint expression

Immune checkpoints such as programmed cell death 1 (PD1), programmed cell death–ligand 1 (PD‐L1), cytotoxic T‐lymphocyte–associated protein 4 (CTLA4), and lymphocyte activation gene‐3 (LAG3) are essential for tumor immune evasion.^99^, ^100^ Recent studies have suggested that proton therapy affects anti‐tumor immunity by altering immune checkpoint expression. For instance, Chen et al. observed that photon therapy increased PD‐L1 expression in HCC cells both in vitro and in vivo.^85^ Although an increase in PD‐L1 was positively correlated with the RBE in HCC,^85^ this finding has been challenged by other studies. Shukla et al. found that after 8 days of pFLASH (60 Gy/s), the proportion of PD‐1^+^CD3^+^ T cells decreased, accompanied by a reduction in PD‐L1 expression in lung cancer cells.^83^ Therefore, arbitrarily increasing the proton irradiation dose is not recommended because of its effects on immune checkpoint expression.

The TIME, including infiltrating immune cells, cytokines, chemokines, and immune checkpoints, has been illustrated in Figure 1. For immune cells, we mainly focused on T cells, B cells, NK cells, TAMs, and neutrophils. For cytokines and chemokines, we illustrated the alterations in IL‐6 and exosomes. We have also introduced the expression of PD‐L1, which changes dynamically with the dose of proton irradiation. Abbreviations: Tregs, regulatory T cells; CD8^+^ TRMs, CD8^+^ tissue‐resident memory cytotoxic T cells; TAMs, tumor‐associated macrophages; NK Cells, natural killer cells; PD‐1, programmed cell death 1; PD‐L1, programmed cell death ligand 1; TIME, tumor immune microenvironment.

## 7 EFFECTS OF PROTON THERAPY COMBINED WITH IMMUNOTHERAPY

### 7.1 Mechanisms of combination therapy

Radiation therapy has a complicated effect on tumor immunity, which can sometimes suppress anti‐tumor responses, contributing to its limited effectiveness.^89^ Thus, combining radiation therapy with immunotherapy has emerged as a potential solution through their synergistic and abscopal effects.^101^, ^102^, ^103^ Because proton therapy has the following features, it is reasonable to combine it with immunotherapy: (i) Relatively low peripheral lymphocytotoxicity: As described in section 4 on the **Impact of Proton Therapy on Peripheral Blood Immune Cells**, proton therapy exerts a less adverse impact on lymphocyte subpopulations, such as CD4^+^ and CD8^+^ T cells, which are indispensable for an effective immune response.^104^ Therefore, the antitumor effects of proton therapy can be enhanced by activating the immune system. (ii) Relatively strong abscopal effect: Proton therapy predominantly induces necrosis in both tumor cells and immune cells instead of apoptosis, releasing more tumor antigens, which can more effectively activate the immune system and thereby enhance the efficacy of immunotherapies.^56^, ^57^, ^105^ (iii) Complicated TME immune landscape: Both immune‐suppressive (TAM2s, MDSCs, Tregs, and PD‐L1) and immune‐activated (TAM1s, CD8^+^T Cells) cells/molecules in the TME can simultaneously increase following proton therapy. Therefore, combining proton therapy with immunotherapy, such as using immune checkpoint blockades (ICBs), could modulate the TME to enhance immune infiltration and activity.^106^ Chen et al. proved this theory in HCC.^85^

### 7.2 Proton therapy combined with ICBs

ICBs such as PD‐1, PD‐L1, and CTLA‐4 inhibitors have been widely used in cancer treatment (PD‐1 inhibitors: nivolumab; PD‐L1 inhibitors: durvalumab; PD‐1 inhibitors: tremelimumab and ipilimumab). In 2020, Nguyen et al. proposed combining proton therapy with ICBs as a therapeutic strategy for locally advanced HNSCC in old patients.^107^ Several clinical studies have supported this perspective. For example, a case study involving anaplastic pituitary adenoma (APA) showed that treatment with ipilimumab and nivolumab achieved complete remission of the recurred APA after proton irradiation and temozolomide chemotherapy, resulting in no residual tumor burden for 34 months and improved survival rates.^108^ Similarly, in two cases of leptomeningeal disease associated with melanoma, combining proton craniospinal irradiation with nivolumab and ipilimumab extended the OS to 7 months and PFS to 5 months while maintaining acceptable toxicity levels.^109^ However, contradictory results have been reported in one case. Maslov et al. found that a patient with adenoid cystic carcinoma who received IMPT followed by nivolumab treatment died 3 months later, suggesting that the effectiveness of this combined therapy may be overestimated.^110^ In addition to case reports, Kim et al. conducted a phase II trial on recurrent or metastatic squamous cell carcinoma of the head and neck in 2024. Combining durvalumab and tremelimumab with proton therapy increased the overall response rate (ORR) to 30.4%, median OS to 11.1 months (95% CI, 6.5–15.8), and median PFS to 3.7 months (95% CI, 1.6–5.7).^111^ Moreover, ongoing research is evaluating the safety and efficacy of a combination of stereotactic body proton therapy with durvalumab in NSCLC, where the precision of proton therapy can mitigate adverse effects while enhancing the therapeutic window of immunotherapies.^112^ Notably, compared with photon therapy, proton therapy has the potential to reduce the risk of tumor progression during ICB therapy because patients receiving proton therapy have higher lymphocyte counts, which could improve the PFS and duration of response.^113^ Considering that the clinical evidence is insufficient and primarily focuses on a few cancer types, further large‐scale prospective studies are necessary to explore the combination of proton therapy and ICBs across various tumors in clinical practice. Additionally, compared with the combination therapy of photon radiotherapy and ICBs, there is a lack of foundational research on the mechanisms underlying the effect of proton therapy. A mechanistic study was conducted by Chen et al., who discovered that an anti‐PD‐L1 antibody could reverse the suppressive immune landscape induced by proton therapy in HCC. Specifically, there was an increase in tumor‐infiltrating T cells and a decrease in MDSC recruitment within the TME.^85^ The possible combinations of ICBs are summarized in Table 3.

**TABLE 3:** Combined Proton Therapy with Immunotherapy or Chemotherapy across Different Types of Tumors.

Tumor Type	Combined Therapy	Evidence	Research group
HNSCC	Durvalumab, Tremelimumab	Phase II Clinical Trial	Kim et al.^111^
Central Nervous System Germ Cell Tumors	Cisplatin, Cyclophosphamide, Etoposide, and/or Bleomycin	Single Tertiary Center Clinical Trial	Hong et al.^114^
Leptomeningeal Disease associated with Melanoma	Ipilimumab, Nivolumab	Case Reports	Sener et al.^109^
Anaplastic Pituitary Adenoma	Ipilimumab, Nivolumab	Case Reports	Shah et al.^108^
Adenoid Cystic Carcinoma	Nivolumab	Case Reports	Maslov et al.^110^
NSCLC	Durvalumab	Review	Mcmillan et al.^112^
	Cisplatin and Paclitaxel	Phase I Clinical Trial	Contreras et al.^115^
	Platinum‐based Chemotherapy	Phase II Clinical Trial	Hoppe et al.^116^
	Albumin‐Bound Paclitaxel and Cisplatin	Clinical Trial	Jin et al.^117^
ESCA	Fluorouracil, and/or Taxanes, and/or Platinum	Clinical Trial	Abana et al.^118^
HCC	anti‐PD‐L1 antibody	Experimental Study	Chen et al.^85^
Pancreatic Cancer	CAR‐T Therapy (targeting mesothelin)	Experimental Study	Amit et al.^119^
	Capecitabine	Phase II Clinical Trial	Rapp et al.^120^
Medulloblastoma	CAR‐T Therapy (targeting GD2)	Experimental Study	Ni et al.^86^
Hodgkin Lymphoma	ABVD; ABVE‐PC; DECA; IE	Clinical Trial	Hoppe et al.^121^
Intrahepatic Cholangiocarcinoma	GCD	Case Reports	Nosaka et al.^122^

Abbreviations: HNSCC, head and neck squamous cell carcinoma; NSCLC, non‐small cell lung cancer; ESCA, esophageal carcinoma; HCC, hepatocellular carcinoma; CAR‐T, chimeric antigen receptor T cell; PD‐L1, programmed cell death ligand 1; ABVD, adriamycin, bleomycin, vinblastine, and dacarbazine; ABVE‐PC, adriamycin, bleomycin, vincristine sulfate, etoposide, prednisone, and cyclophosphamide; DECA, dexamethasone, etoposide, cisplatin, and cytarabine; GCD, gemcitabine, cisplatin, and durvalumab; IE, Ifosfamide and Etoposide.

### 7.3 Proton therapy combined with chimeric antigen receptor T Cell (CAR‐T) therapy

In 1993, the Israeli scientist Zelig Eshhar introduced the concept of CAR‐T therapy,^123^ which was first applied in a phase I clinical study on ovarian cancer.^124^ The main mechanism of CAR‐T cell therapy involves the use of genetic modifications to enable T cells to specifically recognize and attack tumor antigens. This approach can partially overcome tumor cells' immune escape.^125^ Since then, CAR‐T cell therapy has been extended to the treatment of leukemia, lymphoma, multiple myeloma, and other hematological malignancies.^126^ In recent years, researchers have attempted to apply CAR‐T therapy to modulate the TME in solid tumors by targeting immune checkpoints (PD‐1, CTLA‐4, TIM‐3, LAG‐3, B7‐H3, VISTA, and B7S1), immune suppressive cells (MDSCs, Tregs), immune suppressive cytokines (TGF‐β, IL‐10), tumor vasculature, cancer‐associated stromal cells, reprogramed metabolism and hypoxia.^127^, ^128^ CAR‐T therapy has been successful in HCC^129^ and GBM.^130^ Numerous reviews have suggested that combining radiation therapy with CAR‐T cell therapy may be beneficial.^131^, ^132^, ^133^ Recently, proton radiation combined with CAR‐T cell therapy has emerged as a novel tumor treatment strategy (Table 3). For pancreatic cancer, the combination of proton therapy with CAR‐T therapy targeting mesothelin enhanced treatment effectiveness, as proton therapy significantly increased the expression of mesothelin in tumor cells and subsequently contributed to greater infiltration of CAR‐T cells into the tumor. Furthermore, the reactive TME (promotion of TAM1 polarization and reduction in MDSCs), along with the abscopal effect (upregulation of IFN‐γ levels in the serum) can help inhibit tumor growth.^119^ In the following year, Ni et al. discovered that pFLASH could sensitize medulloblastoma to GD2 CAR‐T cell therapy by improving CAR‐T cell infiltration and activation.^86^ Thus, the combination of proton therapy and CAR‐T cells is a promising direction for future research.

## 8 EFFECTS OF PROTON THERAPY COMBINED WITH CHEMOTHERAPY

Conventional chemotherapy stimulates anti‐tumor immunity in several ways: by initiating the release of immunostimulatory molecules from dying tumor cells, mediating off‐target effects on immune cell populations, and activating the whole‐body immune system.^134^ Correspondingly, the combination of photon radiation with chemotherapy can improve anti‐tumor efficacy through additive effects,^135^ which has been widely utilized in numerous tumors such as NSCLC,^136^ lymphoma,^137^, ^138^ esophageal carcinoma,^139^ and pancreatic cancer.^140^ Given that proton therapy exerts excellent therapeutic effects alongside low radiation toxicity, the combination of proton therapy with chemotherapy has been proposed as a new treatment strategy (**Table** **3**). Early studies have suggested that the main advantage of combining proton therapy with chemotherapy lies in reducing radiation toxicity instead of improving survival outcomes. For example, a large cohort study involving patients with Hodgkin's lymphoma showed that those who received proton therapy following standard chemotherapy experienced no early grade 3 toxicity. Additionally, the early relapse‐free survival rates were similar to those of photon radiation treatment.^121^ Similarly, although combining proton therapy with capecitabine chemotherapy did not significantly improve the 1‐year OS of patients with unresectable, borderline resectable, or medically inoperable pancreatic cancer, it did significantly reduce the risk of surgical or perioperative complications in patients with borderline resectable disease.^120^ In patients with central nervous system germ cell tumors, systemic chemotherapy (cisplatin, cyclophosphamide, etoposide, and/or bleomycin) followed by proton therapy did not result in better 5‐year EFS or OS rates than chemotherapy followed by photon therapy. This was true for both low‐risk (PEFS = 0.457, POS = 0.485) and high‐risk (PEFS = 0.605, POS = 0.392) groups.^114^ Recent studies have indicated that combining proton therapy with chemotherapy can improve the survival outcomes of patients with cancer. For NSCLC, Contreras et al. reported that treatment involving large‐fraction proton beam radiation of 60 Gy delivered over 15 sessions, combined with cisplatin and paclitaxel chemotherapy, resulted in high rates of locoregional control and OS, along with good acute tolerability.^115^ A phase I/II single‐arm nonrandomized prospective multicenter trial also reached similar conclusions. Researchers found that large‐fraction proton therapy, delivering doses ranging from 2.5 to 3.53 Gy per fraction and combined with platinum‐based treatment for stage II or III unresectable NSCLC, resulted in high overall survival rates. Specifically, the OS rates were 89% and 49% at 1 and 3 years, respectively, with PFS rates of 58% and 32%.^116^ Additionally, a retrospective analysis of NSCLC with negative driver genes revealed that combination therapy including proton therapy and chemotherapy (albumin‐bound paclitaxel and cisplatin) provided a survival advantage over X‐ray treatment, particularly in the later stages, which eventually contributed to longer OS by the third year (51.6 ± 4.62 months, 95% CI, 42.5 ∼ 60.7 vs 33.1 ± 1.99 months; 95% CI, 29.2 ∼ 37.1; *P*<0.05).^117^ Based on these findings, proton therapy combined with chemotherapy could not only minimize radiation‐related toxicity in NSCLC but also improve survival outcomes, especially in the late stages. IMPT, an efficient type of proton therapy, when used alongside chemotherapy (fluorouracil, taxanes, and/or platinum), could improve long‐term survival outcomes in patients with locally advanced esophageal cancer (5‐year OS rate: 41.1%; PFS: 34.6%; local regional recurrence‐free survival: 78.1%; distant metastasis‐free survival: 65.0%) and simultaneously reduced the risk of cardiopulmonary toxicity.^118^ Differences in the survival outcomes of the combination of proton therapy and chemotherapy could be attributed to tumor heterogeneity and the relatively small sample size. Thus, large‐scale prospective multicenter clinical studies are necessary to better illustrate these therapeutic effects. In addition, research on the transcriptional profile and immune landscape after combination treatment is worthy of attention.

## 9 CURRENT CHALLENGES AND FUTURE DIRECTIONS

### 9.1 Single Cell RNA‐sequencing and radiotherapy biobanking

Currently, research on proton therapy primarily focuses on bulk RNA sequencing in cancers such as BC, GBM, head and neck cancer, prostate cancer, lung cancer, and pancreatic cancer (Table 1). Because single‐cell RNA sequencing better describes biological changes in different cell types, it has been applied to GBM,^141^ BC,^142^ NSCLC,^143^ and head and neck cancer^144^ during photon therapy. However, the application of single‐cell transcriptomics in proton therapy is limited at present. Using single‐cell RNA sequencing, Shiau et al. discovered that proton therapy was associated with a reduction in lymphatic endothelial cells (ECs) and an increase in reactive endothelial‐to‐mesenchymal transition (EndMT) in PDAC. These findings suggest that incorporating nintedanib, an EndMT‐inhibiting drug, into proton therapy may provide a more effective therapeutic strategy.^145^ Furthermore, as biological data accumulate, establishing radiotherapy biobanks could promote data sharing across multiple centers and prevent duplicated efforts.^146^ In summary, utilizing the single‐cell RNA sequencing and establishing an authentic, large‐sample radiotherapy biobanking could improve the personalization of proton therapy.

### 9.2 Technical challenges in clinical applications

The clinical implementation of proton therapy is subject to several technical challenges that hinder its widespread adoption. These challenges include the high costs of building and maintaining proton therapy facilities, complexity of treatment planning systems, and need for specialized training of medical personnel.^147^ In addition, precise delivery of proton beams requires advanced imaging techniques. Robust quality assurance protocols are essential for accurately targeting tumors while sparing the surrounding healthy tissue. Variability in patient anatomy and tumor characteristics further complicates treatment planning, necessitating the development of more sophisticated computational models and machine learning algorithms to predict treatment outcomes.^148^ Overcoming these technical hurdles is essential to optimize proton therapy and establish it as a standard treatment for various cancers.

### 9.3 High quality clinical research

 Proton therapy is expensive and has strict indications. Therefore, conducting large‐scale clinical trials is challenging. However, multi‐center collaborative research may be a potential solution. Because combining proton therapy with other treatment strategies, such as immunotherapy and chemotherapy, may produce synergistic effects, hospitals should collaborate with pharmaceutical companies to facilitate clinical trials of these combination therapies. By performing multi‐cooperative, large‐sample, prospective, authoritative clinical studies, we may accumulate robust evidence regarding the efficacy and safety of proton therapy across various cancer types, which would ultimately benefit patients with cancer through improved treatment options.

## 10 CONCLUSION

In the evolving field of cancer treatment, proton therapy is a promising modality owing to its high efficacy and low toxicity. This review highlights that proton therapy selectively targets cancer cells, spares healthy tissue, and induces a unique immune response in peripheral blood immune cells, tumor cells, and the TIME. In addition, combining proton therapy with immunotherapy and/or chemotherapy may benefit patients with various types of cancer. We hope that this review will provide a better understanding of the impact of proton therapy on the immune system and shed light on potential combination treatment strategies.

## AUTHOR CONTRIBUTIONS

*Conceptualization*: G.H. and Z.T. *Methodology*: R.L., M.T., M.F., Y.J., G.H., and Z.T. *Investigation*: R.L., M.T., M.F., and Z.T. *Data curation*: R.L., M.T., and Y.J. *Writing – original draft preparation*: R.L., M.T., Y.J., M.F., G.H., and Z.T. *Writing – review and editing*: G.H. and Z.T. *Supervision*: G.H. and Z.T. *Funding*: G.H. and Z.T. All authors have read and agreed to the published version of the manuscript.

## INSTITUTIONAL REVIEW BOARD STATEMENT

Not applicable.

## INFORMED CONSENT STATEMENT

Not applicable.

## CONFLICTS OF INTEREST STATEMENT

The authors declare no conflicts of interest.

## Data Availability

Data are provided to those interested, upon reasonable request.
